# Portable, high speed blood flow measurements enabled by long wavelength, interferometric diffuse correlation spectroscopy (LW-iDCS)

**DOI:** 10.1038/s41598-023-36074-8

**Published:** 2023-05-31

**Authors:** Mitchell B. Robinson, Marco Renna, Nisan Ozana, Alyssa N. Martin, Nikola Otic, Stefan A. Carp, Maria Angela Franceschini

**Affiliations:** 1 Athinoula A. Martinos Center for Biomedical Imaging, Massachusetts General Hospital, Harvard Medical School, Charlestown, MA USA; 2 Bar-Ilan University, Tel Aviv District, Ramat Gan, Israel; 3 Department of Biomedical Engineering, Boston University, Boston, MA USA

**Keywords:** Biomedical engineering, Optical sensors, Optical spectroscopy, Blood flow

## Abstract

Diffuse correlation spectroscopy (DCS) is an optical technique that can be used to characterize blood flow in tissue. The measurement of cerebral hemodynamics has arisen as a promising use case for DCS, though traditional implementations of DCS exhibit suboptimal signal-to-noise ratio (SNR) and cerebral sensitivity to make robust measurements of cerebral blood flow in adults. In this work, we present long wavelength, interferometric DCS (LW-iDCS), which combines the use of a longer illumination wavelength (1064 nm), multi-speckle, and interferometric detection, to improve both cerebral sensitivity and SNR. Through direct comparison with long wavelength DCS based on superconducting nanowire single photon detectors, we demonstrate an approximate 5× improvement in SNR over a single channel of LW-DCS in the measured blood flow signals in human subjects. We show equivalence of extracted blood flow between LW-DCS and LW-iDCS, and demonstrate the feasibility of LW-iDCS measured at 100 Hz at a source-detector separation of 3.5 cm. This improvement in performance has the potential to enable robust measurement of cerebral hemodynamics and unlock novel use cases for diffuse correlation spectroscopy.

## Introduction

Diffuse correlation spectroscopy (DCS) is an established optical technique that allows for the non-invasive measurement of tissue blood flow^1^. Through the measurement of diffusely backscattered light, DCS relates the temporal fluctuations of the collected signals to the motion of blood cells through the vasculature. Bedside clinical blood flow monitoring^2^, especially cerebral blood flow monitoring^3^, has exploded as a use case for DCS, with DCS having been used to estimate metrics of cerebral perfusion during surgical procedures^4–8^, cerebral autoregulation^9,10^, cerebrovascular reactivity^11^, intracranial pressure^12–14^, and critical closing pressure^15,16^. While a number of studies including DCS monitoring have been demonstrated in adult populations, due to limitations in cerebral sensitivity and signal-to-noise ratio^17^, the standard DCS technique is better suited to measure blood flow in neonates and children, where the extracerebral tissue (scalp and skull) is significantly thinner than in adults^18,19^. To improve the performance of DCS in adult populations, many groups have developed modifications on DCS which provide improvements to cerebral sensitivity, signal-to-noise ratio, or both. These methods include interferometric detection^20–25^, parallelized speckle detection^26–28^, acousto-optic modulation^29–31^, pathlength resolved methods^32–37^, speckle contrast methods^38–40^, and long wavelength approaches^41,42^. Recent work in our group has shown the utility of the use of long wavelength DCS applied at 1064 nm, though in practice for clinical measurements, currently available commercial detectors either do not have reasonable noise performance for measurements sensitive to deep flow (InGaAs/InP single-photon avalanche diodes (SPADs))^43^ or are too bulky to be applied clinically (superconducting nanowire single-photon detectors (SNSPD)). To address this gap in detector technology, we have developed long wavelength, interferometric DCS (LW-iDCS), which takes advantage of all the benefits of working at 1064 nm and sidesteps the negative aspects of detector technologies sensitive to light at 1064 nm using interferometric detection together with a highly parallel line scan camera sensor (inspired by the work done at shorter wavelengths by Zhou et al.^21,44^). In this work, we directly compare the performance of LW-DCS and LW-iDCS in a pilot human subject study to verify the equivalence of blood flow estimation by the new LW-iDCS technique and compare the quality of the measured signals.

## Methods

### Traditional (homodyne) diffuse correlation spectroscopy (DCS)

Diffuse correlation spectroscopy estimates the flow in tissue through the analysis of the normalized intensity autocorrelation function, ${g}_{2}\left(\tau \right)$. The autocorrelation of the detected signal is connected to the dynamics of the tissue by the Siegert relation^45^, expressed in Eq. (1),

1 $${g}_{2}\left(\tau \right)=1+\beta {\left|{g}_{1}\left(\tau \right)\right|}^{2},$$

where ${g}_{1}\left(\tau \right)$ is the normalized electric field, temporal autocorrelation function and β is the coherence parameter^46^, which is related to the coherence length of the source, the geometry of the measurement, number of modes detected, and the degree of environmental light contamination. The Siegert relation connects the measured signals to the underlying fluctuations of the electric field due dynamic scattering events. The electric field autocorrelation function in the DCS measurement can be described as an integral of individual pathlength-specific correlation functions over the pathlength distribution detected. This form, given in Eq. (2)^47^, allows for the connection between the measured intensity autocorrelation function and the dynamics in the tissue.

2 $${g}_{1}\left(\tau \right)={\int }_{0}^{\infty }P\left(s\right)\mathrm{exp}\left(-\frac{1}{3}{k}_{0}^{2}{n}^{2}\langle \Delta {r}^{2}\left(\tau \right)\rangle \frac{s}{{l}^{*}}\right)ds$$

where P(s) is the distribution of pathlengths, *s*, taken by photons in the tissue, k_0_ is the wavenumber of the detected light in a vacuum, *n* is the index of refraction of the sample, $\langle \Delta {r}^{2}\left(\tau \right)\rangle$ is the mean squared displacement of the scattering particles, and l^*^ is the reduced, mean free path of photons in the tissue which is described as the inverse of the tissue’s reduced scattering coefficient $\left({l}^{*}=\frac{1}{{\mu }_{s}^{^{\prime}}}\right)$. For DCS measurements in tissue, the mean squared displacement term is assumed to reflect diffusive motion^48^ $\left(\langle \Delta {r}^{2}\left(\tau \right)\rangle =6\alpha {D}_{b}\tau =6B{F}_{i}\tau \right)$, where the blood flow index (BF_i_) describes the effective diffusion coefficient, which reflects the true diffusion coefficient (D_b_) multiplied by the probability of scattering events occurring at moving scatterers (α). While this description of flow in vessels as a diffusive process is slightly puzzling, multiple theoretical and simulation studies have examined the appropriateness of the model to describe the detected signals, and found the diffusive process as a good description under standard DCS measurement conditions^48–51^, though some conflicting theories have been proposed^52^. When fitting correlation curves in this study, the model selected for ${g}_{1}\left(\tau \right)$ is that reflecting a semi-infinite sample measured in the reflectance geometry, given in Eq. (3),

3 $${g}_{1}\left(\tau \right)=\frac{{r}_{b}\mathrm{exp}\left(-K\left(\tau \right){r}_{1}\right)-{r}_{1}\mathrm{exp}\left(-K\left(\tau \right){r}_{b}\right)}{{r}_{b}\mathrm{exp}\left(-\sqrt{3{\mu }_{a}{\mu }_{s}^{^{\prime}}}{r}_{1}\right)-{r}_{1}\mathrm{exp}\left(-\sqrt{3{\mu }_{a}{\mu }_{s}^{^{\prime}}}{r}_{b}\right)}$$

where $K\left(\tau \right)=\sqrt{3{\mu }_{a}{\mu }_{s}^{\mathrm{^{\prime}}}+6{k}_{0}^{2}{n}^{2}{\mu }_{s}^{\mathrm{^{\prime}}2}B{F}_{i}\tau }$, μ_a_ is the optical absorption coefficient, ${r}_{1}=\sqrt{{\rho }^{2}+{{l}^{*}}^{2}}$, ρ is the distance between the source and detector, ${r}_{b}=\sqrt{{\rho }^{2}+{\left({l}^{*}+2{z}_{b}\right)}^{2}}$, ${z}_{b}=\frac{2}{3{\mu }_{s}^{\mathrm{^{\prime}}}}\frac{\left(1+{R}_{eff}\right)}{\left(1-{R}_{eff}\right)}$, and ${R}_{eff}\left(n\right)=-1.440{n}^{-2}+0.71{n}^{-1}+0.668+0.0636n$.

### Interferometric (heterodyne) diffuse correlation spectroscopy (iDCS)

To improve the signal-to-noise ratio of blood flow estimates made by DCS, our group and others have implemented DCS system which utilize interferometric detection^20–24^. These approaches are attractive as they allow for an intrinsic improvement to the signal-to-noise ratio of DCS derived blood flow signals^53^ as well as allow for the use of less sensitive, nosier detectors. In this study, we utilize a Mach–Zehnder interferometer, which combines the diffusely scattered light from the sample with a reference signal split from the laser. This results in a form of ${g}_{2}\left(\tau \right)$ which is different than the homodyne ${g}_{2}\left(\tau \right)$, and is given in Eq. (4),

4 $${g}_{2}\left(\tau \right)=1+\beta \frac{{\langle {I}_{S}\rangle }^{2}}{{\langle {I}_{T}\rangle }^{2}}{\left|{g}_{1}\left(\tau \right)\right|}^{2}+\beta \frac{2\langle {I}_{S}\rangle \langle {I}_{R}\rangle }{{\langle {I}_{T}\rangle }^{2}}\left|{g}_{1}\left(\tau \right)\right|,$$

where $\langle {I}_{S}\rangle$ is the average light intensity collected from the sample, $\langle {I}_{R}\rangle$ is the average light intensity of the reference arm of the interferometer, and $\langle {I}_{T}\rangle$ is the average total light intensity falling on the detector.

### Description of optical instrumentation

**Figure 1:**
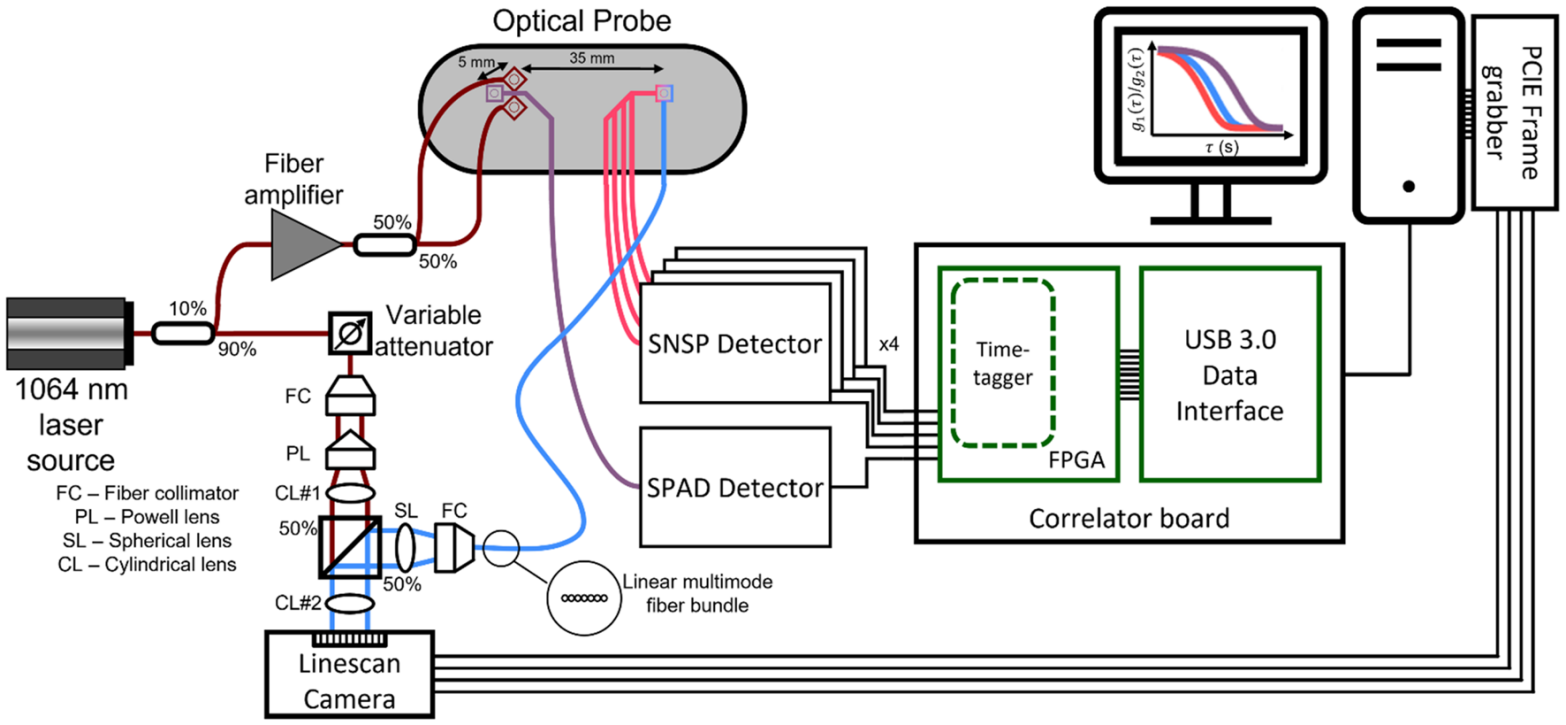
Optical instrumentation used in this work. A long coherence, 1064 nm laser was coupled to a 90%/10% fused fiber coupler to split the light into a reference arm for the interferometer (90%) and the seed source for the fiber amplifier (10%). The amplified source light was split by a 50%/50% fused fiber coupler to supply two MPE limited sources. Single mode fibers were placed at 5 mm (1) and at 35 mm (4) to bring the light to the SPAD detector and the SNSPDs, respectively. The single photon detection events were time tagged at 6.67 ns resolution and transferred to the computer via USB 3.0. Multimode fibers were also placed at 35 mm (7), which brought light to the sample arm of the interferometer. Light from both the reference and sample arms was shaped to match the size of the linescan camera array (12.5 µm × 25.6 mm), and the intensity signals from the camera were digitized at 300 kHz.

A graphical representation of the optical instrumentation is given in Fig. 1. To make direct, collocated comparisons between homodyne LW-DCS and heterodyne LW-iDCS, a custom fiber optic probe was constructed, similar to the probe reported previously^42^, to deliver light from the laser and return light to the detectors. The fiber optic probe contained two adjacent source fibers (> 3.5 mm center-to-center distance), 1 single mode fiber for short-separation DCS (5 mm) and several co-localized long-separation detection fibers: 4 single mode fibers (LW-DCS), and 7 multimode detection fibers (LW-iDCS). A high coherence (l_c_ > 10 km), fiber (MFD 6.6 µm) laser source emitting ~ 125 mW at 1064 nm (RFLM-125-0-1064, NP Photonics) was fusion spliced (S185HS Fusion Splicer, Fitel) to a 90:10, polarization maintaining fused fiber coupler (MFD 6.6 µm, PN1064R2A1, Thorlabs). The 10% arm of the coupler was used as the input for a fiber amplifier (MAKO-AMP1064, Cybel), and was connected via an FC/APC connector. The amplifier output fiber (MFD 10 µm) was fusion spliced to the input of a 50:50, 105 µm, multimode fused fiber coupler (TW1064R5A1B, Thorlabs). The two outputs of the fiber coupler were spliced to two 105 µm multimode source fibers connected to the probe. The light was amplified to allow for two MPE limited spots^54^ (1 W/cm^2^ at 1064 nm, 3.6 mm spot size diameter, 102 mW each spot) to increase the achievable signal-to-noise ratio. The 90% output arm of the polarization maintaining coupler was connected to the reference arm input of the LW-iDCS interferometer. All spliced connections were confirmed by the fusion splicer to have losses less than 0.03 dB.

For the homodyne LW-DCS measurements, a custom LW-DCS detection system based on single-photon detectors [one silicon SPAD (SPCM-NIR-14, Excelitas) and four SNSPDs tuned to 1064 nm (Opus One, Quantum Opus)] and a custom FPGA-based software correlator board with a timing resolution of 6.67 ns was used^55^. Single mode detector fibers were used to bring the backscattered light from the optical probe to the detectors. The silicon SPAD was used to detect light collected at a source-detector separation of 5 mm (i.e. short separation), and the four SNSPDs were used to detect light collected at a source-detector separation of 35 mm (i.e. long separation). For the short separation signal, autocorrelation functions were calculated at a rate of 10 Hz due to the limited photon counts available. For long separation signals, autocorrelation functions for each channel were calculated at a rate of 100 Hz and subsequently averaged.

For the heterodyne LW-iDCS measurements, a free space Mach Zehnder interferometer was constructed to project the light from both the sample and reference optical fibers onto a fast InGaAs linescan camera (Manx 2048 SQ CXP 390, Xenics). The 90% arm of the polarization maintaining coupler was connected to a variable optical attenuator (VOA1064-APC, Thorlabs), which was connected to a fiber collimator (F220APC-1064, Thorlabs) placed in a kinematic mount providing XY translation (CXY1A, Thorlabs) as well as tip and tilt (KAD11F, Thorlabs). A Powell lens (LGL130, Thorlabs) was used to homogenize the intensity of the reference arm along the dimension of pixel array of the linescan camera. The diverging output of the Powell lens was collimated by a cylindrical lens (LJ1765L1-C, Thorlabs). For the sample arm, the seven 200 µm, multi-mode fibers in the optical probe placed at a source-detector separation of 35 mm were aligned linearly and bundled into an SMA connector (FG200LEA, BFL200LS02, Thorlabs) and collimated using a fiber collimator (F220SMA-1064, Thorlabs). To correct the remaining angular divergence in the sample arm, a spherical lens (LA1131-B-ML, Thorlabs) was used. Both the sample and reference beams were sent to a 50:50 non-polarizing beam splitter (CCM1-BS014, Thorlabs) and the combined output was focused onto the linear array of the camera using a cylindrical lens (LJ1328L2-B, Thorlabs). The ratio of sample arm intensity to reference arm intensity during the measurements was estimated to be 1 to 5 × 10^7^. This was estimated by scaling the estimated average power per fiber mode at 3.5 cm at 1064 nm (~ 1.2 × 10^−12^ mW per source fiber (~ 6 kcps)^42^) by the number of modes contained in the 7 detector fibers (~ 59,000) and dividing by the total reference arm power used (~ 6 mW). The interferometer was built on an optical breadboard (MBH1224, Thorlabs) with vibration isolating feet (AV4, Thorlabs) and placed on a rolling cart (61 × 46 × 122 cm^3^) for mobility. The collected signals were digitized at a line rate of 300 kHz. Raw data were captured by a frame grabber (Coaxlink Quad G3, Euresys) and saved directly to disk at a rate of 1.2 GB/s and post processed to estimate BF_i_. Several signal processing steps were completed to maximize measurement SNR and remove camera induced distortions prior to converting the recorded pixel intensity data into autocorrelation functions. These steps included processing to address hardware induced distortions caused by the integrate-while-read mode, quadratically detrending the signals over the analysis interval, averaging adjacent pixel signals, and removal of common noise signals across the camera. These steps are detailed both graphically and in text in the supplement. Correlation functions from the LW-iDCS instrument were computed at a rate of 100 Hz. For multilayer tissue, in the process of assessing the simulated performance of the different DCS implementations, it was discovered that if the semi-infinite model is used to fit the data and if the same percentage of the decay is used for fitting (i.e. correlation function decays to 5% of the value of the plateau), the sensitivity of the measurement to the cerebral signal differs between DCS, based on fitting a function that is proportional to ${\left|{g}_{1}\left(\tau \right)\right|}^{2}$, and iDCS, based on fitting a function that is proportional to $\left|{g}_{1}\left(\tau \right)\right|$. To address this, LW-iDCS correlation functions, proportional to ${g}_{1}\left(\tau \right)$, are fit with a weighted fitting approach to match the cerebral sensitivity of the LW-DCS measurement, which reflect a correlation function based on ${\left|{g}_{1}\left(\tau \right)\right|}^{2}$. The applied weighting approach was optimized using a Monte Carlo simulation of light transport and momentum transfer in a multi-layer, slab geometry, detailed in Table S1, that is meant to represent the typical tissue geometry of a measurement made over the forehead.

### Human subject experiments

For this study we enrolled five healthy subjects (3 female, 2 male, age 38 ± 19, included participants of Middle Eastern (1), European (2), Southeast Asian (1), and East Asian (1) descent, 2 with dark skin pigmentation) to compare and validate the performance of the LW-iDCS system against the standard 1064 nm DCS system across several physiological manipulations. This study was reviewed and approved by the Mass General Brigham Institutional Review Board (#2019P003074). All participants gave written informed consent prior to the measurements. All methods were performed in accordance with the relevant guidelines and regulations. The measurement protocol included three tasks: breath holding, hyperventilation, and tourniquet pressure modulation.

Due to the lack of head coverage in this study, systemic physiological manipulations were selected to induce large, repeatable changes in the measured blood flow. Breath holding and hyperventilation were performed to perturb heart rate^56^, blood pressure^57,58^, and vasoactive state^59,60^ to cause changes in both scalp and brain blood flow. The tourniquet pressure modulation technique is used to selectively reduce the blood flow in the scalp to increase the specificity of the blood flow signal to cerebral blood flow^61^. By comparing the reduction in blood flow at the short separation channel and the long separation channel, this method also allows the for the assessment of the long channel’s sensitivity to the cerebral signal. A list of the timing of the activity and recovery intervals can be seen below in Table 1. In addition to the LW-DCS optical instrumentation, systemic physiological monitoring was performed, and included electrocardiography (ECG), pulse oximetry (SpO_2_), continuous, non-invasive blood pressure monitoring (Nova, Finapres), and respiratory monitoring. These signals were digitized at 1 kHz by a Powerlab ADC (ADInstruments). Data collection was synchronized between the measurement devices using an external trigger box which was connected to an auxiliary analog input channel of the LW-DCS FPGA correlator and a channel on the Powerlab ADC. Rough synchronization for the LW-iDCS instrument was accomplished by providing a trigger to the other instruments when the LW-iDCS acquisition was started. Fine tuning of the synchronization was accomplished by determining the time offset between the LW-DCS and LW-iDCS around the trigger which maximized the cross-correlation between the BF_i_ signals.

**Table 1:** Description of the timing intervals of the human subject experiments.

Experiment paradigm	Timing information
Breath holding	60 s baseline, 4 x (30 s end expiratory breath hold, 40 s normal breathing), 20 s recovery
Hyperventilation	60 s baseline, 60 s hyperventilation @ 70 breaths/min, 120 s recovery
Tourniquet pressure modulation	60 s baseline, 3 x (30 s probe pressure, 30 s release), 60 s recovery

### Comparison of blood flow measured during physiologic manipulations

To compare the blood flow changes during the physiological manipulations, we first removed the cardiac pulsation from the blood flow signals. To remove the influence of the cardiac pulsatility, using the previously identified RR intervals from the ECG, averaged beat-to-beat blood flow index values were calculated^62^. The data were then resampled back to the original sampling rate (i.e. 100 Hz for long separation signals, 10 Hz for short separation signals), and the periods of physiological manipulation were identified and separated. To compare between trials and between subjects, individual trial BF_i_ values were scaled to a relative blood flow index (rBF_i_) by dividing by the average BF_i_ in the 20 s preceding the activity. The individual trials for each experimental paradigm were then averaged across subjects. 

## Results

### Optimization of the weighted fitting approach to compare between iDCS and DCS for multi-layer tissue

**Figure 2:**
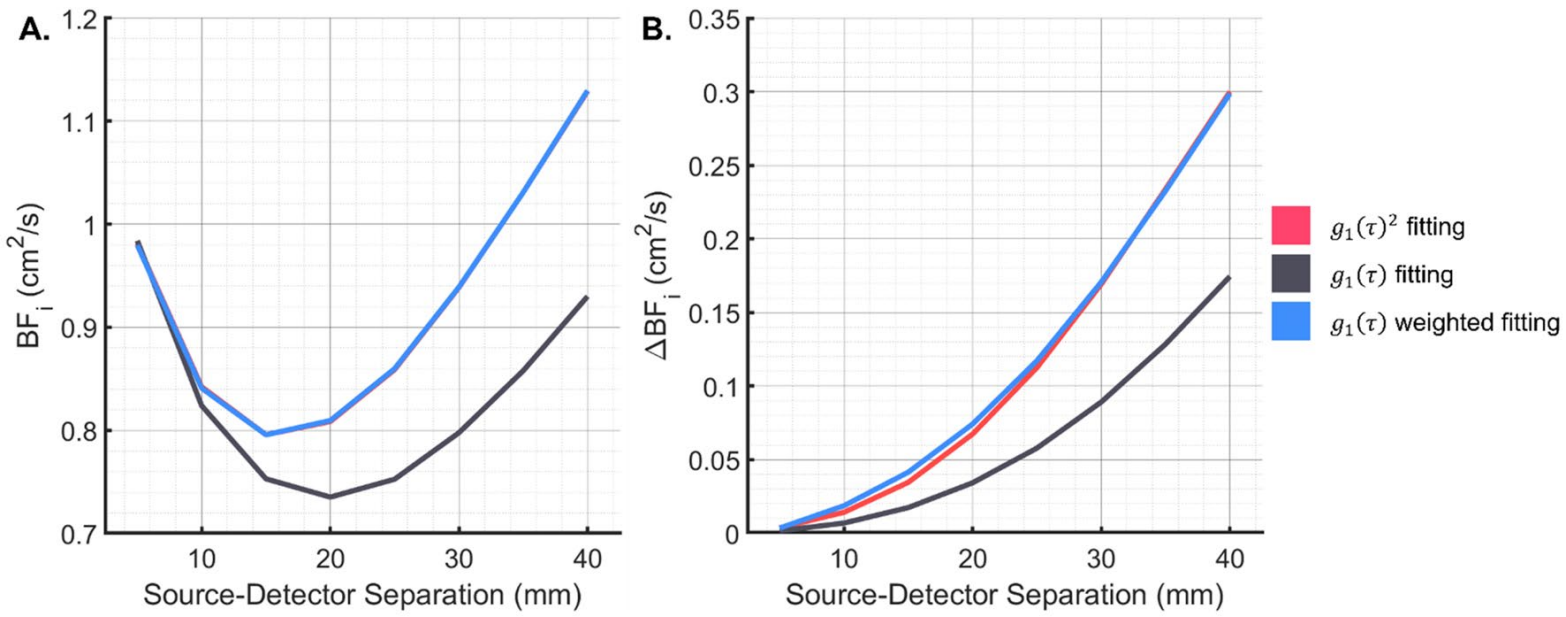
Comparison of the BF_i_ fit from simulated multilayer DCS measurements. (**A**) The BF_i_ fit from the baseline simulations with different fitting functions can be seen. The discrepancy between the fits of ${g}_{1}\left(\tau \right)$ and ${g}_{1}{\left(\tau \right)}^{2}$ would be observed as discrepancies between the fits of iDCS and DCS, respectively. (**B**) The change in BF_i_ measured in response to a 50% increase in the brain BF_i_ is shown. Without the weighted fitting, the iDCS measurement resolves ~ 50% of the changes that DCS does, reducing the sensitivity to the cerebral signal. With weighted fitting, the fit based on ${g}_{1}\left(\tau \right)$ is seen to be equivalent to the fit based on ${g}_{1}{\left(\tau \right)}^{2}$.

To investigate the difference in measured BF_i_ between correlation functions proportional to $\left|{g}_{1}\left(\tau \right)\right|$ and ${\left|{g}_{1}\left(\tau \right)\right|}^{2}$, a baseline and activated condition (+50% brain BF_i_) are simulated^63^. The observed difference in the baseline BF_i_ and sensitivity to cerebral blood flow changes between iDCS and DCS simulations of the three-layer geometry can be seen in Fig. 2A and B. One considered method to make the two measurements equivalent was to square the collected iDCS correlation function to have a signal proportional to ${\left|{g}_{1}\left(\tau \right)\right|}^{2}$. While on noise-less correlation functions, this would be the preferred method, with noisy correlation functions, taking the square of the curves could result in distortions to the noise properties of the curves, which could further affect the fitting. The alternative approach that was taken was to increase the weighting given to the earlier part of the correlation function using a data driven weighting scheme. The optimized objective function is given in Eq. (5), where the weighting coefficients are taken from the overall average correlation function from the entire measurement.

5 $$obj=\sum {\left({g}_{1,Measured}\left(\tau \right)-{g}_{1,Model}\left({BF}_{i},\tau \right)\right)}^{2}*{\langle {g}_{1,Measured}\left(\tau \right)\rangle }_{T}^{x}$$

where the ${\langle \rangle }_{T}$ is the average over the measurement interval, and *x* is a factor that was optimized to reach equivalent BF_i_ from the simulations. A range of factors, x, were investigated using the simulated data from the comparison of DCS and iDCS performance. Based on the tissue geometry simulated, the optimal value was found to be 2.5, and this value was fixed and used to fit the data in the manuscript. The corrected baseline blood flow and resolved changes can be seen in Fig. 2A and B.

### Characterization of the equivalency of the measured blood flow and comparison of the noise performance between LW-DCS and LW-iDCS when resolving cerebral blood flow signals

**Figure 3:**
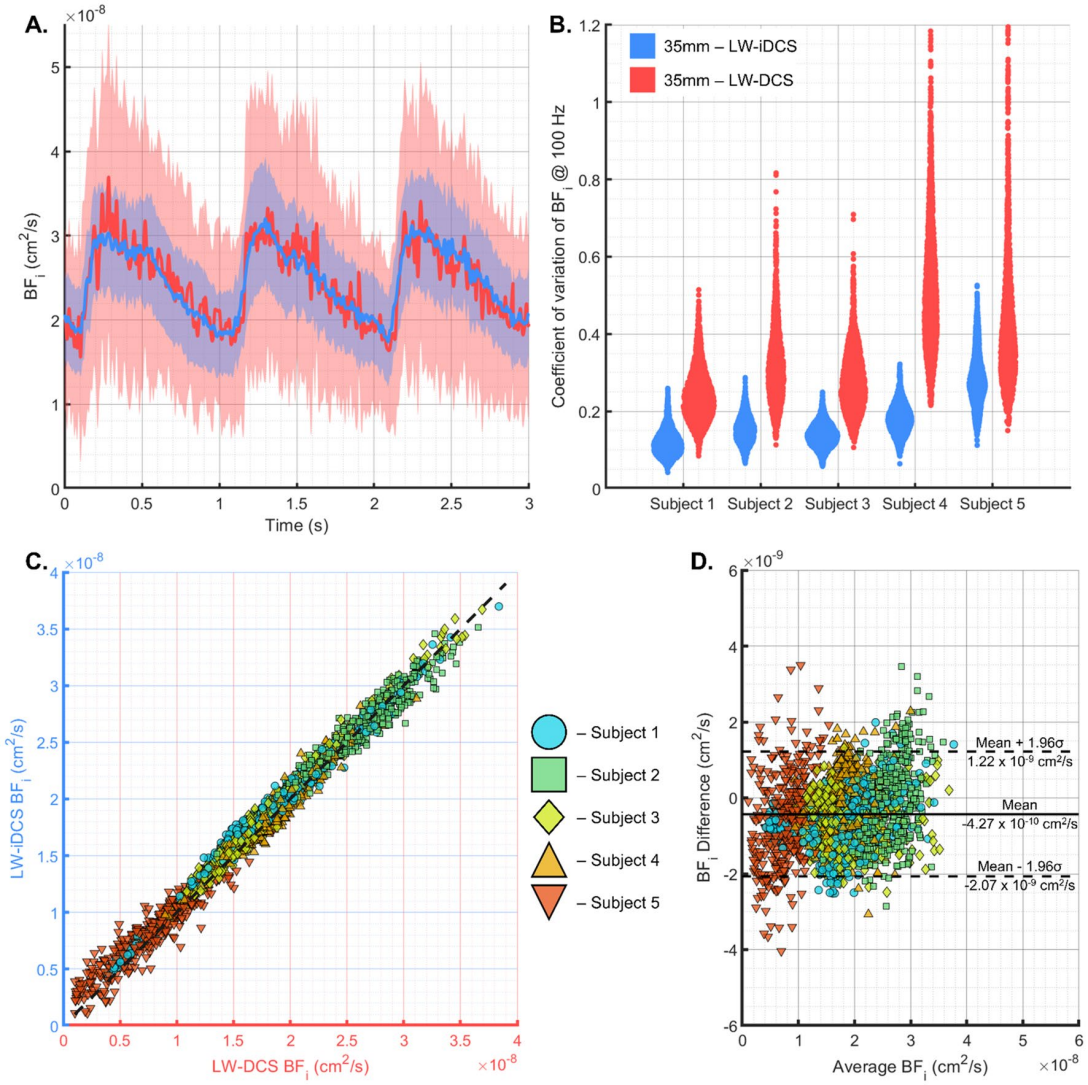
Comparison of the characteristics of BF_i_ time traces measured at 100 Hz from the LW-iDCS and LW-DCS instruments. (**A**) An example of a single subject, pulsatile cardiac signal is shown for both instruments, demonstrating equivalency in the measured blood flow index as well as the reduced noise of the blood flow trace measured by the LW-iDCS instrument. (**B**) The coefficient of variation $\left({\sigma }_{B{F}_{i}}/{\mu }_{B{F}_{i}}\right)$ was computed for each point in the cardiac cycle, and the results for each subject for each measurement modality are shown in violin plots. On average, the reduction in coefficient of variation provided by the LW-iDCS instrument is ~ 2.25× when compared to the 4 channel LW-DCS instrument. Equivalency of the measured BF_i_ values beyond the pulsatile signals between the two instruments is also shown across subjects and tasks using the cardiac filtered, BF_i_ signals. (**C**) The measured BF_i_ values are plotted against each other, and cluster nicely around the line of unity. (**D**) The Bland–Altman plot shows a narrow distribution of the differences in the measured BF_i_, characterized by a mean difference of 4.27 × 10^−10^ cm^2^/s and a standard deviation of 8.39 × 10^−10^ cm^2^/s, demonstrating good agreement between the two blood flow measurements.

To assess the equivalency of the measurements taken by the LW-iDCS instrument, blood flow traces are compared between the LW-DCS and LW-iDCS instruments. To assess both noise performance as well as equivalence of the BF_i_ measured for the pulsatile signal, cardiac gated averaging was performed using data from the baseline periods where the variability in the shape of the cardiac pulsation and modification in heart rate can be assumed to be minimal. RR intervals were identified in the time aligned ECG traces, and LW-DCS and LW-iDCS BF_i_ values were averaged in sync with the cardiac cycle, as was done previously^15^. In Fig. 3A, the time aligned blood flow trace measured by each instrument is shown for three cardiac cycles (n = 20 averaged three-cycle traces) in an example subject with the standard deviation of the estimates of blood flow shown as well. The equivalence of measured blood flow index is also confirmed across all experimental conditions and subjects. Average BF_i_ values were calculated for each cardiac pulse identified in all subject BF_i_ time traces across all tasks, and the BF_i_ values from each instrument are plotted against each other in Fig. 3C, showing excellent agreement over the range of measured BF_i_. In Fig. 3D, a Bland–Altman plot comparing the agreement of the measured BF_i_ between the two instruments shows a bias (4.27 × 10^−10^ cm^2^/s) for the LW-iDCS instrument to measure a faster blood flow with a standard deviation of the difference equal to 8.39 × 10^−10^ cm^2^/s. The bias and spread of the difference are relatively small compared to the range of values typically measured with DCS (Fig. 3C), and this result indicates good agreement between the two instruments. To assess the noise performance across subjects, the coefficient of variation of the measured pulsatile BF_i_ at each point in the cardiac cycle is calculated for each instrument. These values are compared as violin plots in Fig. 3B and show an average reduction in the coefficient of variation given by LW-iDCS of ~ 2.25×. This matches quite well with the estimated improvement in contrast-to-noise ratio (CNR) estimated from Monte Carlo simulation, shown in the supplement.

### Subject averaged response to breath holding

**Figure 4:**
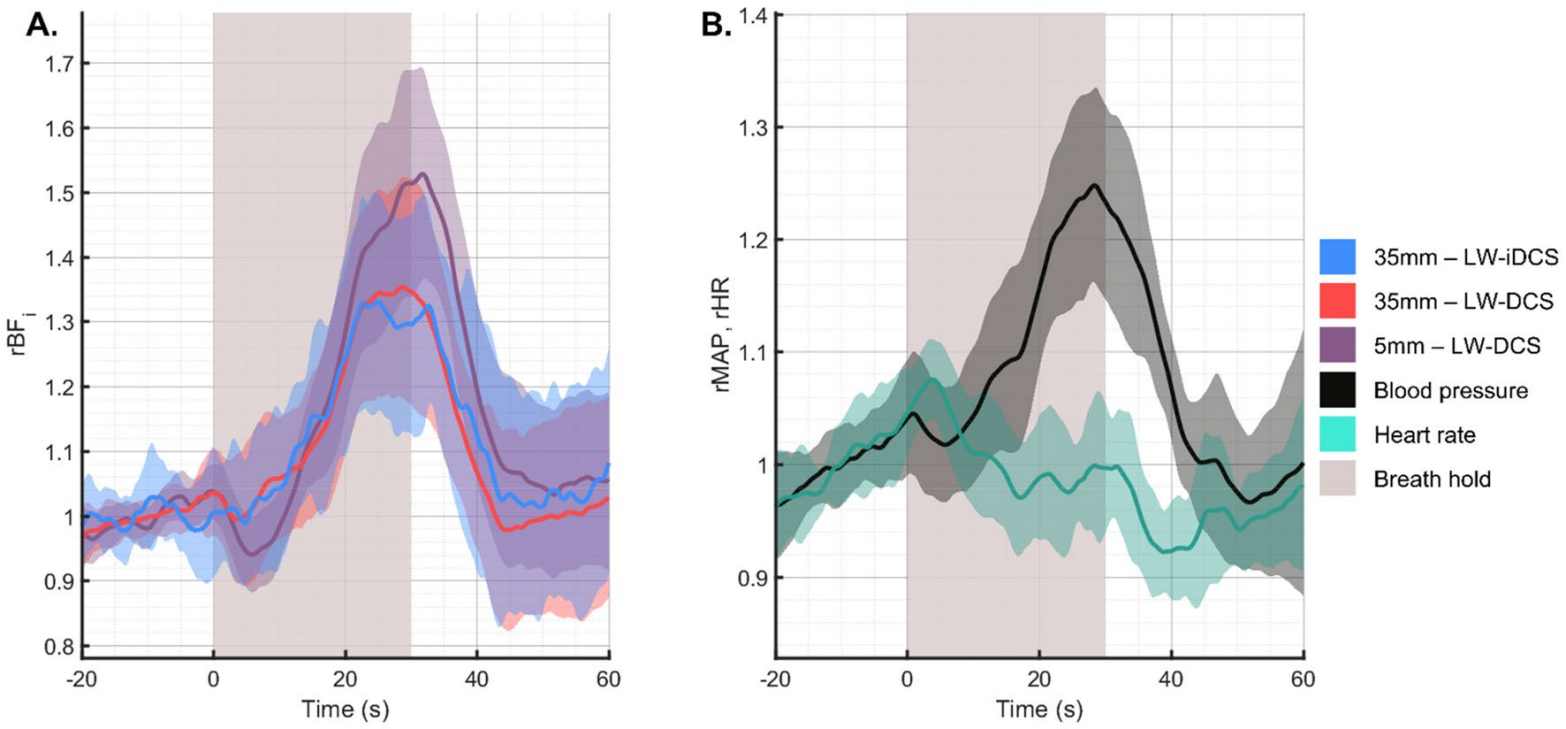
Subject averaged responses to breath holding. (**A**) Comparison of the measured blood flow responses to the 30 s breath hold. The relative change in flow in the long separation measurements is seen to be slightly lower than the change observed at the short separation, which has been previously observed^42^. (**B**) Comparison of relative changes in blood pressure and heart rate, respectively in response to the 30 s breath hold. A progressive increase in blood pressure was observed throughout the breath holding period, while the heart rate remains relatively constant.

In response to an end expiratory breath hold, typical physiological responses include an increase in blood pressure^57,58^ and a hypercapnic state^64,65^. In Fig. 4, we show the subject averaged response to a 30 s breath holding trial. The mean arterial pressure (MAP) is seen to increase 25% ± 9% by the end of the breath hold, while the heart rate remains relatively constant. The BF_i_ increases measured at the long separation are matched well between LW-DCS and LW-iDCS (32% ± 17% at end breath hold) and are seen to be distinct from the increase measured at the short separation (51% ± 17% at end breath hold). The observed increases in blood flow are consistent with the expectation given an increase in blood pressure and the mild hypercapnic state.

### Subject averaged response to hyperventilation

**Figure 5:**
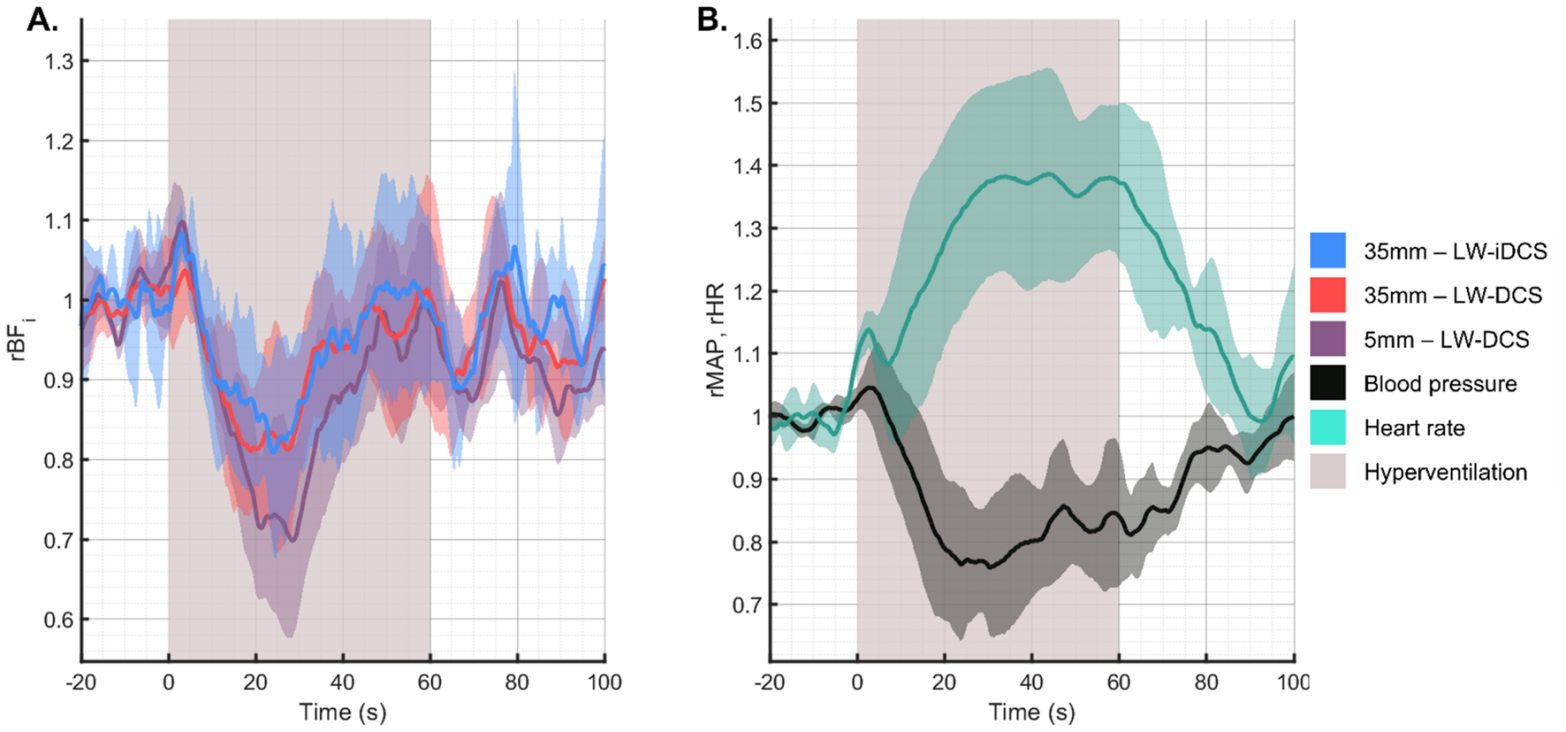
Subject averaged response to hyperventilation maneuver. (**A**) Measured hemodynamic response to 60 s of hyperventilation. As in the breath holding task, the short separation measurement shows a more exaggerated response to the physiologic manipulation, exhibiting a decrease of 30% in BF_i_ after the onset of hyperventilation. The matching long separation responses show a lesser degree of BF_i_ reduction, and all blood flow can be seen to return to the baseline before the end of the hyperventilation trial. (**B**) For this maneuver, the heart rate increased significantly following the start of the trial, while the blood pressure was seen to reduce.

For the hyperventilation task, subjects performed one minute of paced breathing at seventy breaths per minute. The expected hypocapnia due to the over breathing is expected to cause vasoconstriction and a decrease in blood flow. Because cerebral and scalp tissue metabolism is maintained during this period, the reduced blood flow results in a decrease in tissue hemoglobin saturation (SO_2_), which induces a vasoactive reaction to return the blood flow to the baseline level^59^. This biphasic response was observed in both the long and short channel measurements, as seen in Fig. 5A. The observed systemic physiologic response to the bout of hyperventilation was a significant increase in the heart rate (38% ± 15%) and a significant decrease in the mean arterial pressure (−18% ± 10%), as seen in Fig. 5B. The latency of the return to baseline of these physiologic parameters was longer as compared to the latency of the return to baseline of the measured blood flow. The long separation blood flow signal responses to hyperventilation were seen to be consistent, and additionally match well to the responses observed from the long separation blood flow measurements previously reported in our group^42^.

### Subject averaged response to tourniquet pressure modulation

**Figure 6:**
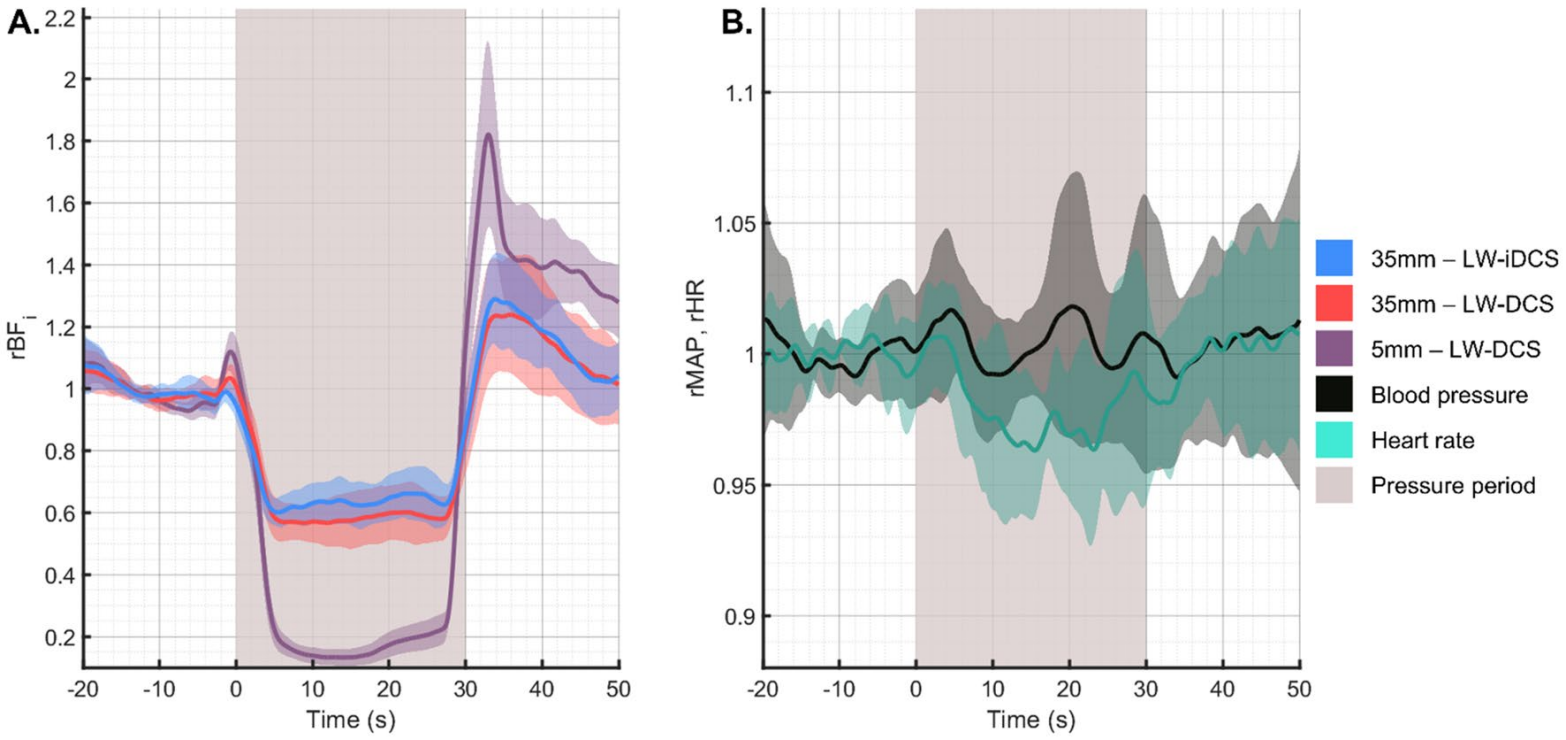
Subject averaged response to the pressure modulation maneuver. (**A**) Measured hemodynamic response to 30 s of tourniquet tightening. Using the ratio of the relative decrease between the long channel (39.2%) and the short channel (85.3%), the sensitivity of the long channel to the scalp blood flow can be estimated to be 46%. For long separation DCS measurements, brain sensitivity has been shown to be inversely proportional to scalp sensitivity^66^, and we can estimate that the 35 mm separation measurement has a brain sensitivity > 50%. (**B**) For this maneuver, as expected, the systemic physiology was not significantly affected by the tightening of the tourniquet on the forehead.

The tourniquet tightening causes selective reduction of blood flow in the scalp, leaving the brain blood flow unmodified^61^. By comparing the differential response between a short separation channel, sensitive almost exclusively to the blood flow in the scalp, and a long separation channel, exhibiting sensitivity to both the scalp and cerebral hemodynamics, estimates of the long separation channel’s sensitivity to each compartment can be estimated. The group averaged blood flow traces from the pressure modulation trials can be seen in Fig. 6A. In this group of subjects, the average reduction in BF_i_ measured was 85.3% and 39.2% for the short and long channels, respectively. This pair of measurements corresponds to a long separation channel with a 46% sensitivity to the superficial blood flow signal, which implies a cerebral sensitivity of over 50%, based on a recent simulation study^66^. As was expected for this maneuver, the systemic physiology did not respond to the change in torniquet pressure, seen in Fig. 6B.

## Discussion

In this work we have demonstrated the development of long wavelength, interferometric diffuse correlation spectroscopy. Using a fiber optic probe with collocated detection fibers, we were able to directly compare measured blood flow and signal-to-noise ratio between the LW-DCS and LW-iDCS instruments. For both single photon detection and interferometric detection, the availability of cost-effective, high-power fiber optic amplifiers, an engineering benefit of making measurements at 1064 nm, allows for an increased SNR beyond the intrinsic benefits of using light at 1064 nm. By sacrificing spatial resolution, multiple sources spaced > 3.5 mm apart could be used, allowing for an even higher SNR for high quality pulsatile blood flow measurements. The SNR of the LW-iDCS measurement seen in the high-speed pulsatile measurements was 4.5× the SNR of the SNSPD LW-DCS measurement when making single channel comparisons, representing an enabling improvement to the quality of blood flow measured. In the context of the DCS systems currently used for translational research, this improvement is especially significant considering that even the single illumination SNSPD LW-DCS has an SNR gain of 16× over conventional DCS^42^, and that measurements at 3.5 cm are not feasible with conventional NIR DCS. The use of a camera which is sensitive to light at 1064 nm takes advantage of both the higher number of photons per mode as compared to traditional NIR wavelengths as well as the slower decay of the autocorrelation function. For cerebral blood flow measurements made at long source-detector separations, the autocorrelation decay for traditional NIR DCS can happen in 1–10 s of microseconds, and a significant portion of the decay could be missed if not sampled quickly enough. The use of both heterodyne detection, measuring the slower decaying $${g}_{1}\left(\tau \right)$$ as opposed to $${g}_{2}\left(\tau \right)$$, and 1064 nm relaxes the sampling rate needed to effectively sample the correlation function. The longer source-detector separation achievable with these advanced DCS systems enables measurements with reduced sensitivity to the upper tissue layers relative to the sensitivity of currently applied DCS systems in the traditional NIR wavelength range (explored in the supplement). The decreased sensitivity to extracerebral signals is greatly beneficial to DCS measurements, especially in clinical applications where systemic physiological fluctuations are more likely to occur and the timing of relevant cerebral hemodynamic changes is not as well defined. We also see good agreement with the estimated noise performance given by Monte Carlo simulation (Figure S3). Additionally, the cost of the system is greatly reduced compared to LW-DCS based on SNSPDs. For this implementation of the LW-iDCS system, the detector used is ~ 7× less expensive (~ $25 k total, camera + frame grabber: ~ $20 k, assorted lenses, opto-mechanics, and fibers: ~ $5 k) as compared to the SNSPDs (~ $180 k total, cryostat: ~ $100 k, individual nanowire detectors: ~ $20 k each). The LW-iDCS cart-based system is also more mobile than the SNSPD based LW-DCS system. These improvements in cost, SNR, and mobility are promising for the clinical usability of LW-iDCS measurements of CBF in adults. The signal processing approach used to extract the correlation function from the raw data stream points to potential pitfalls in the development of iDCS instruments using multimode fiber and free space interferometers though. The motion of fibers and vibrations in the environment have the potential to corrupt the iDCS signals, however, these challenges are manageable, and the use of the custom data analysis pipeline, described in supplementary information, was successful in removing artifacts from the data. The use of a weighted fitting approach allowed for equivalent blood flow indices to be fit from both the LW-DCS and LW-iDCS correlation functions, evidenced by the results shown in Fig. 3C and D. While the results presented matched well, investigation of the generalizability of the weighting factor selected in this study is warranted given the influence that tissue layer thicknesses, optical properties, and ratios of scalp and brain blood flow are known to have on fitting autocorrelation functions^67,68^. Another challenge posed by the implementation of massively parallel multi-speckle detection is the raw data rate of the instruments. Recent publications on massively parallelized detection have quoted raw data rates between 0.24 GB/s (0.864 TB/hr) and 9.0 GB/s (32.4 TB/hr)^22,25–28,44,69^. For clinical blood flow measurements, these data rates could result in untenably large data files, though real time processing utilizing GPUs or FPGAs have been explored as a solution to address this challenge^28,69^. The increased SNR provided by the LW-iDCS instrument presented here enabled high sensitivity to the cerebral blood flow signal as well as a high rate of BF_i_ calculation. These factors will be highly enabling for the clinical translation of DCS as a noninvasive cerebral blood flow monitor.

## Supplementary Information

Supplementary Information.

## Data Availability

The data that support the findings of this study are available from the corresponding authors upon reasonable request.
