# Supplementary material for: Portable, high speed blood flow measurements enabled by long wavelength, interferometric diffuse correlation spectroscopy (LW-iDCS)
Source: Sci Rep. 2023 May 31;13:8803. doi: 10.1038/s41598-023-36074-8 (PMC10232495; doi:10.1038/s41598-023-36074-8)
Supplement: Supplementary file 1 — Supplementary Information. [file 41598_2023_36074_MOESM1_ESM.docx]

Portable, high speed blood flow measurements enabled by long wavelength, interferometric diffuse correlation spectroscopy (LW-iDCS), supplementary information

**Mitchell B. Robinson^1,*^, Marco Renna^1^, Nisan N. Ozana^1,2^, Alyssa N. Martin^1^, Nikola Otic^1,3^, Stefan A. Carp^1^, Maria Angela Franceschini^1^**

^1^Athinoula A. Martinos Center for Biomedical Imaging, Massachusetts General Hospital, Harvard Medical School, Boston, MA, USA

^2^Bar-Ilan University, Ramat Gan, Tel Aviv District, Israel

^3^Department of Biomedical Engineering, Boston University, Boston, MA, USA

*Corresponding Author: Mitchell Robinson ([mitchell.robinson@mgh.harvard.edu](mailto:mitchell.robinson@mgh.harvard.edu))

## Description of data analysis procedure for LW-iDCS

To account for the noise sources introduced by the camera, environment, and laser source, we devised a custom data analysis pipeline to denoise the collected intensity traces prior to the calculation of the temporal autocorrelation function. In this work, correlation functions were calculated at 100 Hz (0.01 s). Pixel intensity signals are given as $I[n,p]$, where n is temporal sample index, and p is the pixel index from 1 to 2048. The step-by-step pipeline is given below:

1. Correct individual pixel intensity signals for high frequency oscillations induced by the different ADC offset of the two ADCs used to enable the integrate-while-read (IWR) mode. For this camera and others with similar architecture, the acquisition speed is increased by utilizing two individual ADC modules to allow the camera to simultaneously read from the previous integration time, $I[n-1,p]$, while sampling the current signal intensity, $I[n,p]$. This difference in hardware introduces a high frequency oscillation, as alternating lines are captured by different ADCs. To correct for this, we perform temporal averaging of *M* alternating lines, and subtract the averages from the appropriate even or odd numbered lines, i.e. $DC_{even}\left[ p \right]=\frac{1}{M}\sum_{m=0}^{M} I\left[ 2m+2,p \right]$ and $DC_{odd}\left[ p \right]=\frac{1}{M}\sum_{m=0}^{M} I[2m+1,p]$. Correction of the high frequency oscillation is done in this way and not with a low pass filter which eliminates only the frequency $\frac{f_{s}}{2}$ to prevent the loss of information from the first bin of the correlation function. The gap between acquired frames is also corrected. For the camera settings used in this study, the interframe interval is exactly the length of one line acquisition, so interpolation between the edges of the frames is used to compute the signal in between frames.
2. Following the correction of the high frequency oscillation, the intensity signals are quadratically detrended over the sampling period (0.01s). This is done to reduce the influence of laser intensity drift or possible signal drifts caused by motion of the optical setup.
3. To improve individual pixel signal-to-noise ratio, we follow the approach described previously by Zhou^1^, and apply a gaussian blur across the pixel array, taking advantage of the physical size of individual speckle signals and the correlated intensity fluctuations between pixels. For this system, we optimized the size of the gaussian kernel by comparing the signal-to-noise ratio of the collected correlation function at different blurring kernel widths, shown in **Figure S1**.
4. Singular value decomposition (SVD) is performed on the data to identify common signals across pixels. Before passing the intensity signals to the SVD algorithm, pixel signals are mean subtracted and divided by their temporal standard deviation, an operation equivalent to taking the temporal z-score of the signal. High rank components, i.e. common components, are removed from the decomposed matrices, and the signals are reconstituted. This step addresses signals that are common to many pixels, which do not contribute to the spatially discrete, time varying speckle signals of interest. In this study, the ten highest ranked components were removed during the SVD step^2^.
5. Pixel-wise calculation of the autocorrelation is performed, and these individual pixel autocorrelations are averaged.

Additionally, to further account for the noise introduced by the reference arm and the environmental vibrations, a “no source” measurement is also taken. This is accomplished by turning off the optical amplifier while the probe is still attached to the forehead of the subject. The measurement characterizes noise sources that are present in the reference arm as well as the effect of environmental vibration on the projection of the reference arm to the camera. Equation (4) in the main text is derived under the assumption that the reference intensity has a constant value correlation function and does not contribute to the shape of the measured correlation function. Under realistic conditions, fluctuations in the reference power will introduce a temporal structure to the reference arm intensity, which necessitates a correction to allow for the accurate assessment of BF_i_. Here we will describe the correction method, where a correlation function calculated from the “no source” measurement is subtracted from the full measurements to yield an accurate representation of the electric field, autocorrelation function, $g_{1}\left( \tau\right)$. We first describe the electric field of the intensity modulated, reference arm, given as,

$$\begin{aligned} E_{R}\left( t \right)=\sqrt{{\langle I}_{R}\rangle+\Delta I_{R}\left( t \right)}e^{-j\phi_{R}},\#\left( S1 \right) \end{aligned}$$

where $\left\langle I_{R} \right\rangle$ is the average reference intensity, $\Delta I_{R}\left( t \right)$ is the intensity fluctuation, with mean equal to 0, standard deviation equal to $\gamma\left\langle I_{R} \right\rangle$, and a normalized intensity correlation function equal to $g_{R}\left( \tau\right)$, $\gamma$ is the coefficient of variation of the reference arm intensity signal, and $\phi_{R}$ is the static phase increment of the reference arm determined by the pathlength through the optical system. We have not included the fast, optical frequency fluctuation of the phase for simplicity. The electric field incident on the detector is a sum of the reference and sample arms, and can be described as,

$$\begin{aligned} E\left( t \right)=E_{S}\left( t \right)+E_{R}\left( t \right),\#\left( S2 \right) \end{aligned}$$

where E_S_(t) is the electric field of the sample arm. The intensity of the combined signal can then be described as,

$$\begin{aligned} I\left( t \right)=\left( E_{S}\left( t \right)+E_{R}\left( t \right) \right)\left( E_{S}^{*}\left( t \right)+E_{R}^{*}\left( t \right) \right)=I_{S}\left( t \right)+I_{R}\left( t \right)+2Re\left[ E_{S}\left( t \right)E_{R}^{*}\left( t \right) \right],\#\left( S3 \right) \end{aligned}$$

where the time dependent, reference intensity can be split into a constant term, $\left\langle I_{R} \right\rangle$ and a perturbation term, $\Delta I_{R}\left( t \right)$. For small values of $\gamma$, we can apply Taylor expansion to the expression describing the reference arm electric field, given by,

$$\begin{aligned} E_{R}\approx\sqrt{\left\langle I_{R} \right\rangle}\left( 1+\frac{\Delta I_{R}\left( t \right)}{2\left\langle I_{R} \right\rangle} \right)e^{-j\phi_{R}}.\#\left( S4 \right) \end{aligned}$$

This simplification allows for the decomposition of both the reference arm intensity term as well as the reference arm electric field term into static and dynamic components, given as,

$$\begin{aligned} I\left( t \right)=I_{S}\left( t \right)+\left\langle I_{R} \right\rangle+2\sqrt{\left\langle I_{R} \right\rangle}Re\left[ E_{S}\left( t \right)e^{j\phi_{R}} \right]+\Delta I_{R}\left( t \right)+\frac{1}{\sqrt{\left\langle I_{R} \right\rangle}}Re\left[ E_{S}\left( t \right)\Delta I_{R}\left( t \right)e^{j\phi_{R}} \right].\#\left( S5 \right) \end{aligned}$$

From the form of equation (S5), the terms that give rise to the noiseless correlation expression given in equation (4) in the main text can be seen as the first three terms of the right-hand side of equation. The last two terms reflect the intensity fluctuations of the reference arm and the heterodyne interference with the fluctuations in the reference arm. The unnormalized, intensity autocorrelation function for this signal is given in equation (S6),

$$\begin{aligned} G_{2}\left( \tau\right)=\left\langle I_{S} \right\rangle^{2}+2\left\langle I_{S} \right\rangle\left\langle I_{R} \right\rangle+\left\langle I_{R} \right\rangle^{2}+\beta\left\langle I_{S} \right\rangle^{2}\left| g_{1}\left( \tau\right) \right|^{2}+2\beta\left\langle I_{S} \right\rangle\left\langle I_{R} \right\rangle\left| g_{1}\left( \tau\right) \right|+\left\langle I_{R} \right\rangle^{2}\gamma^{2}g_{R}\left( \tau\right)+\frac{\beta}{2}\left\langle I_{S} \right\rangle\left\langle I_{R} \right\rangle\gamma^{2}g_{R}\left( \tau\right)g_{1}\left( \tau\right).\#\left( S6 \right) \end{aligned}$$

The form of this correlation function is again organized where the terms that give rise to the noiseless description of the autocorrelation function are listed first, and the perturbative terms are listed after. For the “no source” measurements, the form of the detected correlation function is given in equation (S7),

$$\begin{aligned} G_{2}\left( \tau\right)=\left\langle I_{R} \right\rangle^{2}{+\left\langle I_{R} \right\rangle}^{2}\gamma^{2}g_{R}\left( \tau\right).\#\left( S7 \right) \end{aligned}$$

Making the assumptions that (1) the reference intensity does not vary between the time the “no source” measurement is taken and when the real measurements are taken and that (2) the correlation function of the reference arm, $g_{R}\left( \tau\right)$, also does not vary with time, if the result of the “no source” measurement is subtracted from the result of the actual measurement, a large possible noise source will be removed. Given in equation (S8), the results of the subtraction show the removal of the larger of the two perturbative terms, which is proportional to the square of the reference intensity. For further simplification, with a reference arm dominated measurement, $\frac{I_{R}}{I_{T}}\approx1$, terms that are proportional to the square of the average sample intensity will be negligible and are removed.

$$\begin{aligned} G_{2}\left( \tau\right)=2\left\langle I_{S} \right\rangle\left\langle I_{R} \right\rangle+2\beta\left\langle I_{S} \right\rangle\left\langle I_{R} \right\rangle\left| g_{1}\left( \tau\right) \right|+\frac{\beta}{2}\left\langle I_{S} \right\rangle\left\langle I_{R} \right\rangle\gamma^{2}g_{R}\left( \tau\right)g_{1}\left( \tau\right)\#\left( S8 \right) \end{aligned}$$

The subtraction of the “no source” correlation function removes one noise term, though the modulated $g_{1}\left( \tau\right)$ is still present in the expression. The inclusion of the modulated correlation function in this expression is not so bothersome though, as the time constant of the reference arm correlation function, $g_{R}\left( \tau\right)$, typically corresponds to vibrational noise sources in the environment that are relatively low frequency (10 Hz – 1kHz)^3^. This frequency range will provide a slower time constant than the time constant of the electric field autocorrelation function at long source-detector separations, and the product of the two functions will be dominated by $g_{1}\left( \tau\right)$. Further, because the coefficient of variation of the laser source intensity used for iDCS is typically small (for the system described in this work, $\gamma<0.5\%$), the amplitude of the modulated correlation function will be quite small and will be dominated by the uncorrupted term proportional to $g_{1}\left( \tau\right)$. BF_i_ is then fit from the “no source” subtracted measurements. The entire processing pipeline can be seen graphically in **Figure S2**.


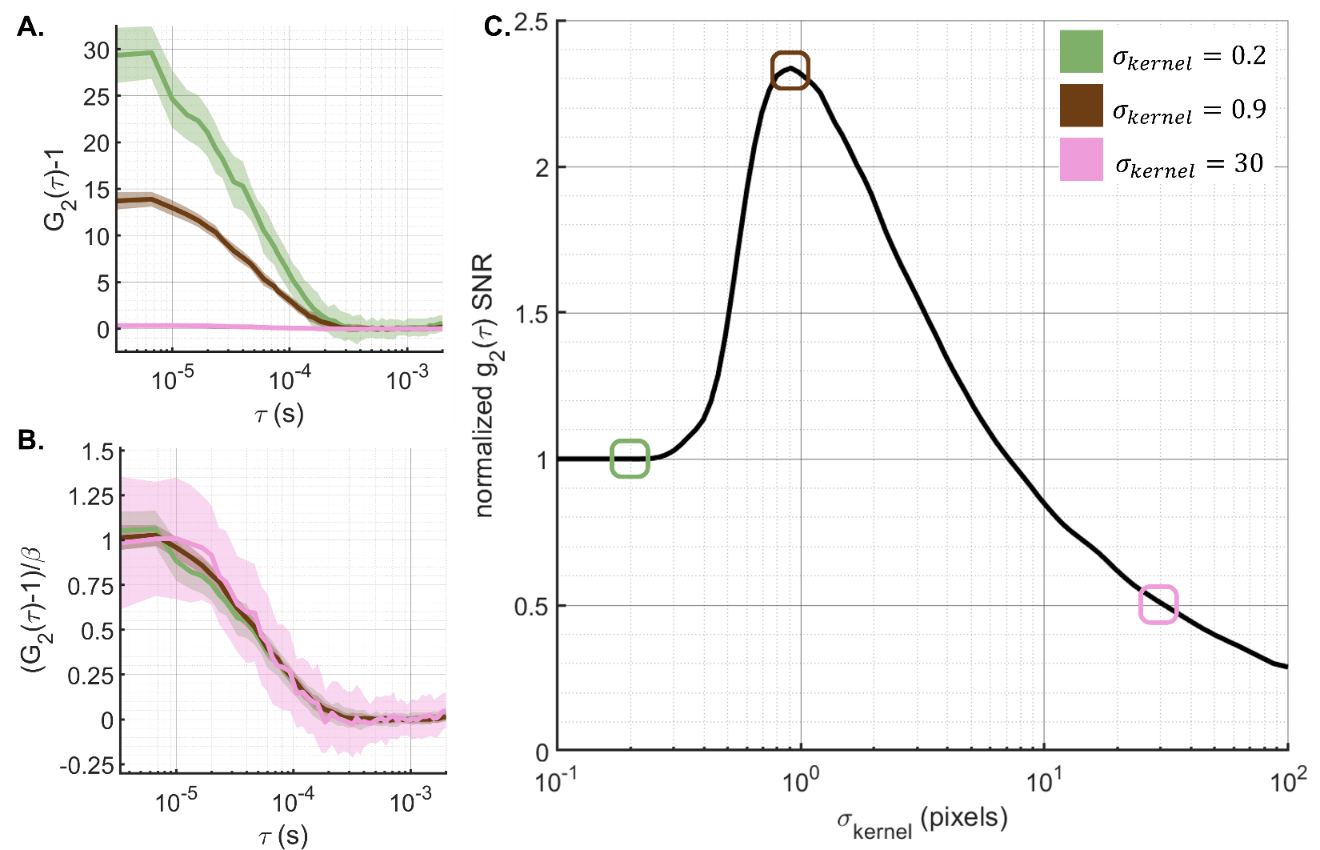


**Figure S1.** Comparison of the signal to noise ratio of the computed autocorrelation as a function of the size of the blurring kernel used. The curves seen in (A) and (B) correspond to the similarly colored working points labeled in (C). In (A) the curves are left unnormalized, and the amplitude of the correlation function can be seen to be higher for the smaller blurring kernels. In (B) the correlation functions are normalized by the value at zero lag, where the signal-to-noise ratio of the curves are easier to compare visually. For the plot in (C), the average signal-to-noise ratio of the first 50% of the decay of the curve is computed for each size of blurring kernel. The optimal kernel size parameter for this optical setup is 0.9, which corresponds to a full-width half maximum size of 2.1 pixels.


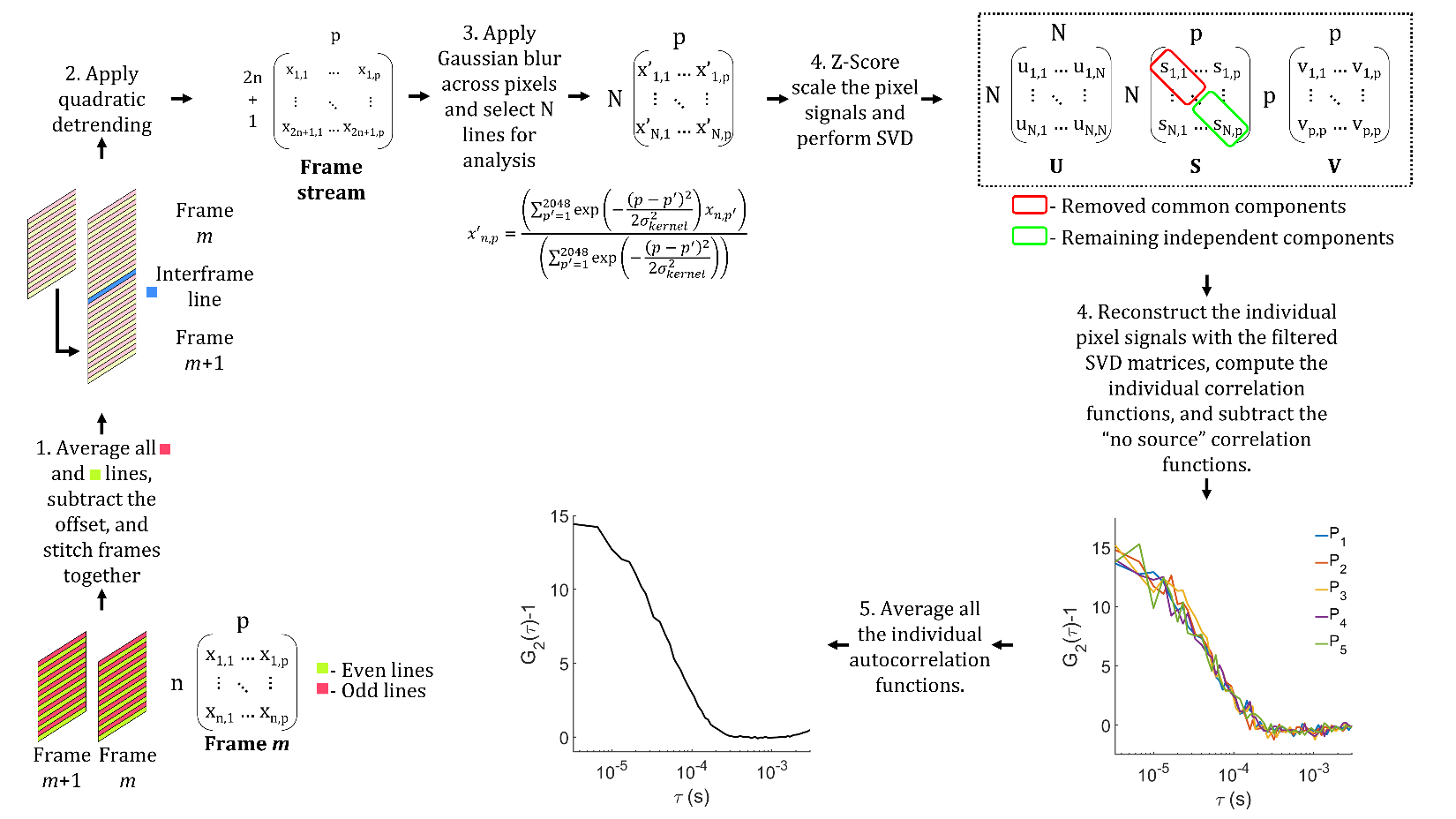


**Figure S2.** Visual description of the signal processing steps discussed above.

## Monte Carlo Simulation of DCS performance between 850 nm DCS, 1064 nm DCS, and 1064 nm iDCS

Monte Carlo simulations were performed to compare the expected sensitivity and noise properties of different DCS implementations. A three-layer, slab tissue model approximating the scalp, skull, and brain was simulated using MCX^4^. The layer thicknesses, optical properties, and blood flow values are given in **Table S1**. Source-detector separations between 5 mm and 40 mm with 5 mm spacing were simulated. Electric field autocorrelation functions, $g_{1}\left( \tau\right)$, were calculated for both 850 nm and 1064 nm at each simulated source-detector separation for both a baseline condition and a condition where the brain blood flow was simulated to be 50% greater using equation (S9).

$$\begin{aligned} g_{1}\left( \tau\right)=\frac{\sum_{all photons} \exp\left( \frac{1}{6}\sum_{i=1}^{n} Y_{i}\left\langle\Delta r_{i}^{2}\left( \tau\right) \right\rangle\right)\exp\left( -\sum_{i=1}^{n} l_{i}\mu_{a,i} \right)}{\sum_{all photons} \exp\left( -\sum_{i=1}^{n} l_{i}\mu_{a,i} \right)}\#\left( S9 \right) \end{aligned}$$

Where *Y_i_* is the momentum transfer accumulated for a given photon trajectory in medium *i*, *l_i_* is the pathlength of a given photon trajectory in medium *i*, and $\mu_{a,i}$ is the medium specific absorption coefficient. To compare between the different implementations of DCS, a contrast-to-noise index (CNR_i_) was used, defined as the sensitivity of the measurement to changes in the brain blood flow divided by the coefficient of variation of the fitted BF_i_ with the appropriate noise added. Sensitivity to brain blood flow was defined as the relative change in measured blood flow divided by the relative change in the brain blood flow, i.e. $\mathrm{Sensitivity}=\frac{BF_{i,perturbed}-BF_{i,baseline}}{BF_{i,baseline}*50\%}$. Because the sensitivity of the measurement to deeper flow is typically higher when shorter correlation lags are used^5^, several sets of fitting indices were used starting at time 0 and stopping at $\tau_{fit}$. To assess the noise of the measurement, the simulated curves are made noisy by adding noise consistent with the expected photon count rate, correlation function decay rate, and averaging time using the correlation noise expression derived by Koppel^6^, given in equation (S10),

$$\begin{aligned} \sigma\left( \tau\right)=\sqrt{\frac{T}{t}}\left[ \beta^{2}\frac{\left( 1+e^{-2\Gamma T} \right)\left( 1+e^{-2\Gamma\tau} \right)+2\frac{\tau}{T}\left( 1-e^{-2\Gamma T} \right)e^{-2\Gamma\tau}}{1-e^{-2\Gamma T}}+2\left\langle n \right\rangle^{-1}\beta\left( 1+e^{-2\Gamma\tau} \right)+\left\langle n \right\rangle^{-2}\left( 1+\beta e^{-\Gamma\tau} \right) \right]^{\frac{1}{2}}\#\left( S10 \right) \end{aligned}$$

where $\sigma\left( \tau\right)$ is the noise at a given time lag, τ; T is the width of the correlation function time bin; t is the averaging time of the measurement; β is the coherence parameter of the measurement; Γ is the decorrelation rate, modeling the autocorrelation function as $g_{2}\left( \tau\right)=1+\beta\exp\left( -2\Gamma\tau\right)$; and $\left\langle n \right\rangle$ is the number of photon counts within a correlation time bin of width T. Decay rates of the correlation functions (Γ) were estimated by fitting a single exponential model for $g_{1}\left( \tau\right)$. For the iDCS simulations, modifications to the noise model are performed consistent with the recommendations given in Koppel^6^ for coherent light scattering measurements made in the heterodyne configuration. These modifications include (1) $\Gamma\to\frac{\Gamma}{2}$, reflecting the slower decorrelation of $g_{1}\left( \tau\right)$, (2) $\left\langle n \right\rangle\to\left\langle n_{T} \right\rangle$, where $\left\langle n_{T} \right\rangle$ is the sum of the sample arm count rate, $\left\langle n_{S} \right\rangle$, and the reference arm count rate, $\left\langle n_{R} \right\rangle$, and (3) $\beta\to\beta_{0}\frac{2\left\langle n_{S} \right\rangle}{\left\langle n_{T} \right\rangle}$, where β_0_ is the coherence parameter of the equivalent homodyne measurement. The ratio of $\frac{\left\langle n_{S} \right\rangle}{\left\langle n_{T} \right\rangle}$ was set equal to 2/10^8^, reflecting the ratio seen in a reference arm dominated measurement. We expect that due to the reference arm dominating the detected light, the primary noise source will be shot-noise, as opposed to dark or read noise, which will allow for a reasonable prediction of the noise properties of the signal using this model even with the non-photon counting detector. Photon count rates are estimated based on subject averaged, in-vivo count rates from a previous study^7^ (11 kcps per source fiber for 850 nm @ 25 mm and 114 kcps per source fiber for 1064 nm @ 25 mm) and scaled based on the intensity distribution as a function of source-detector separation determined by the Monte Carlo simulation and the photon detection efficiency of each of the simulated detectors. The coupling efficiency of the sample arm in the iDCS system is also incorporated in the scaling of the detected photon count rate^8^. The computed noise for each simulated detector was then scaled by the square root of the number of independent speckle observations (NIO) made (NIO = 4 for 850 nm DCS, NIO = 4 for 1064 nm DCS, NIO = 890 for 1064 nm iDCS). The number of independent observations for the iDCS instrument is calculated using the FWHM of the size of the speckle on the camera, determined from the peak of the Gaussian blurring curve in **Figure S1.C**, the number of pixels in the camera, and the relationship describing the number of independent observations given these two parameters^9^. 50 noisy realizations of the baseline condition for each source-detector separation were generated and fit over a range of correlation lags from [0,$\tau_{fit}$] to compute a coefficient of variation as a function of the $\tau_{fit}$. The CNR_i_ for each source-detector separation was computed as a function of the fitting range, and the maximum value of the CNR_i_ is saved and plotted. As was seen previously^10^, in each case fitting the entire curve provides the highest CNR­_i_ at each source-detector separation. The comparison of CNR_i_ and brain sensitivity at the maximum CNR_i_ for a measurement made at 1 Hz can be seen in **Figure S3**, comparing between the traditional 4 channel, 850 nm DCS device based on silicon SPADs; the 4 channel, 1064 nm DCS device based on super conducting nanowire detectors; and the 1064 nm iDCS device described here based on the InGaAs linescan camera.

| Table S1: Optical properties and geometry of the simulated tissue used in the Monte Carlo simulations | | | | | | | |
| --- | --- | --- | --- | --- | --- | --- | --- |
| **Tissue Geometry** | **Thickness [mm]** | **μ_a,850_ [cm^-1^]** | **μ_a,1064_ [cm^-1^]** | **μ_s_^’^_,850_ [cm^-1^]** | **μ_s_^’^_,1064_ [cm^-1^]** | **Index of refraction** | **Diffusion Coefficient [cm^2^/s]** |
| Scalp | 5 | 0.164 | 0.11 | 7.4 | 5.3 | 1.4 | 1x10^-8^ |
| Skull | 7 | 0.115 | 0.13 | 8.1 | 5.8 | 1.4 | 1x10^-10^ |
| Brain | Semi-infinite | 0.17 | 0.17 | 1.16 | 8.3 | 1.4 | 6x10^-8^ |


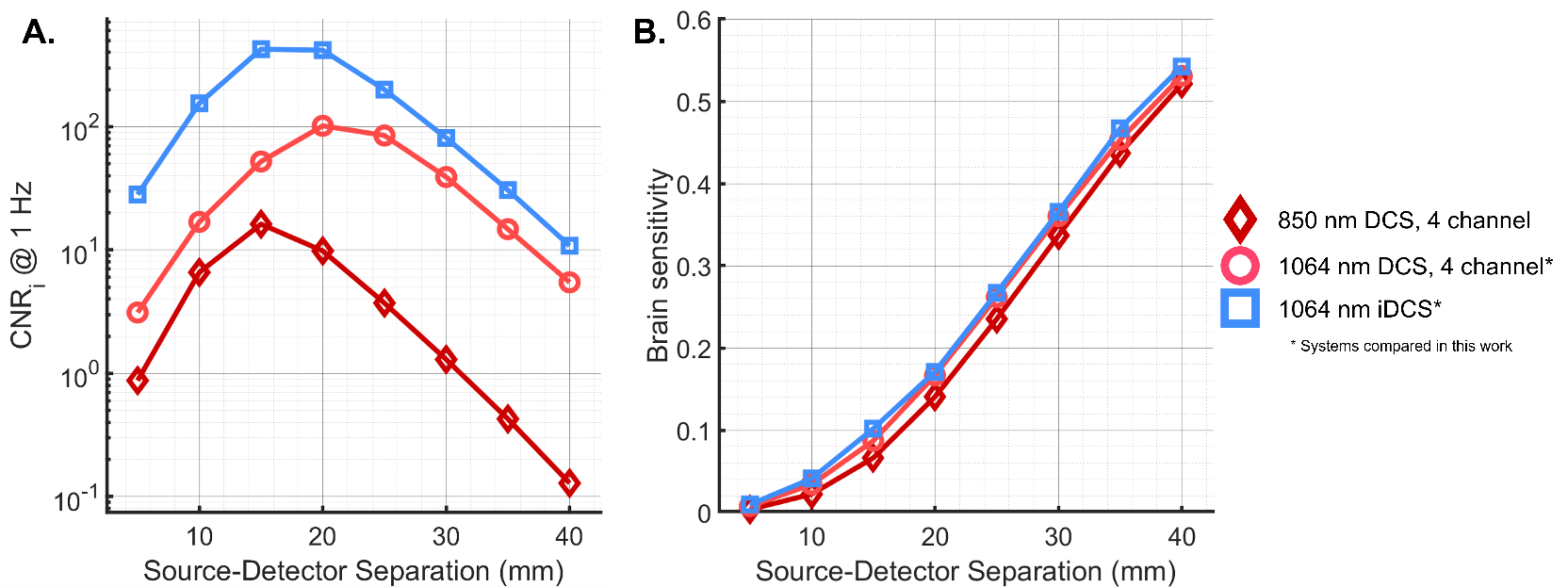


**Figure S3.** Comparison of the simulated performance of different implementations of DCS instruments. In (A) the CNR_i_ for the typical clinical implementation of DCS (4 co-localized detection channels, 850 nm) is compared to the two systems compared in this work. For the 35 mm source-detector separation used in this work, the use of 1064 nm, either with the SNSPD based system or the iDCS system, can be seen to improve CNR_i_ by a factor of >30x. In (B) the sensitivity determined at the maximum CNR_i_ is compared between all implementations. The sensitivity across the range of source-detector separations does not differ by more than 5% between each of the simulated systems, though the slightly higher sensitivity of 1064 nm DCS and 1064 nm iDCS comes because of the higher SNR of the correlation function, and, likely, the better utilization of the earlier correlation lags not as disrupted by noise.

# References

1. Zhou, W. *et al.* Multi-exposure interferometric diffusing wave spectroscopy. *Optics Letters, Vol. 46, Issue 18, pp. 4498-4501* **46**, 4498–4501 (2021).

2. Robinson, M. B., Carp, S. A., Peruch, A., Ozana, N. & Franceschini, M. A. High framerate, InGaAs camera for interferometric diffuse correlation spectroscopy (iDCS) beyond the water peak. in vol. 11641 22 (SPIE-Intl Soc Optical Eng, 2021).

3. Ryaboy, V. M., Kasturi, P. S., Nastase, A. S. & Rigney, T. K. Optical table with embedded active vibration dampers (smart table). in *Proc.SPIE* (ed. White, E. V.) vol. 5762 236 (2005).

4. Fang, Q. & Boas, D. A. Monte Carlo simulation of photon migration in 3D turbid media accelerated by graphics processing units. *Opt Express* **17**, 20178–20190 (2009).

5. Selb, J. *et al.* Sensitivity of near-infrared spectroscopy and diffuse correlation spectroscopy to brain hemodynamics: simulations and experimental findings during hypercapnia. *Neurophotonics* **1**, 15005 (2014).

6. Koppel, D. Statistical accuracy in FCS. *Phys Rev A  (Coll Park)* **10**, 1938–1945 (1974).

7. Ozana, N. *et al.* Superconducting nanowire single-photon sensing of cerebral blood flow. *Neurophotonics* **8**, 35006 (2021).

8. Robinson, M. B. Interferometric, acousto-optic modulated diffuse correlation spectroscopy @ 1064 nm (AOM-iDCS) toward higher sensitivity, non-invasive measurement of cerebral blood flow. (Massachusetts Institute of Technology, 2022).

9. Zilpelwar, S. *et al.* A model of dynamic speckle evolution for evaluating laser speckle contrast measurements of tissue dynamics. *Biomed Opt Express* **13**, 6533–6549 (2022).

10. Carp, S. A., Robinson, M. B., Cheng, X., Boas, D. A. & Franceschini, M. A. Comparing Brain Perfusion Sensitivity between Diffuse Correlation Spectroscopy and Speckle Contrast Optical Spectroscopy. *Society for Functional Near-Infrared Spectroscopy Biennial Meeting, 2022* Preprint at (2022).
